# Ventilator‐Induced Lung Injury: Mechanotransduction and Potential Therapeutic Targets

**DOI:** 10.1002/mco2.70598

**Published:** 2026-02-03

**Authors:** He Ren, Ziqi Shang, Alastair G. Stewart, Ying‐xin Qi, Kai Huang

**Affiliations:** ^1^ Key Laboratory of Biomechanics and Mechanobiology, Ministry of Education, School of Biological Science and Medical Engineering, Beihang University Beijing China; ^2^ Institute of Mechanobiology & Medical Engineering, School of Life Sciences & Biotechnology Shanghai Jiao Tong University Shanghai China; ^3^ Department of Biochemistry and Pharmacology University of Melbourne Parkville Victoria Australia; ^4^ ARC Centre for Microphysiological Systems (MiPSET) Melbourne Australia

**Keywords:** inflammation, mechanical stress, reactive oxygen species, ventilator‐induced lung injury

## Abstract

Mechanical ventilation (MV) serves as a critical intervention to maintain adequate gas exchange. Unfortunately, MV often leads to the development of ventilator‐induced lung injury (VILI). VILI pathogenesis involves alveolar‐capillary barrier disruption, dysregulated inflammation, and mechanotransduction‐driven cellular dysfunction, but the interplay of these mechanisms remains incompletely understood. Here, we review the types of mechanical stress in VILI, key signaling pathways implicated in MV‐induced lung injury, with particular emphasis on the impact of altered mechanical forces in VILI. Furthermore, we discuss the cell‐specific mechanisms in VILI. We also delineate the intricate molecular mechanisms that orchestrate intercellular communication in VILI. In addition, we discuss the limitations of current clinical strategies, and the identification of novel drug targets with transformative potential for treatment of VILI. Moreover, we summarize the current and emerging therapeutic strategies and discuss the existing knowledge gaps and future directions for VILI prevention. By integrating mechanical mechanistic insights with translational perspectives, this review identifies novel biomarkers and potential therapeutics to mitigate VILI. Our synthesis not only advances the understanding of VILI pathophysiology but also provides a framework for precision medicine approaches in critical care, ultimately optimizing MV outcomes.

## Graphical abstract

Mechanical ventilation (MV) is expected to undergo significant transformative advancements by 2050, with the emergence of personalized ventilation systems that promise to revolutionize respiratory care. However, the risk of ventilator‐induced lung injury (VILI) remains a significant concern at this stage and VILI still exists at that time. Therefore, we propose therapeutic approaches based on the fundamentals of mechanobiology. They include drug pre‐treat before MV, exploring the mechanobiological mechanisms in VILI, elucidating the common lung injury mechanisms between LPS and MV and finding more biomarkers and therapeutic targets accordingly.

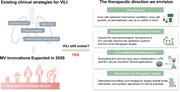

## 1 Introduction

Modern mechanical ventilation (MV) originated from Andreas Vesalius' work five centuries ago. It is a machine‐based therapeutic intervention to assist with the work of breathing that is commonly employed to address conditions that cause either low oxygen levels or high carbon dioxide levels [1]. It is a life‐saving intervention [2] required in some specific conditions due to severe respiratory or neurological dysfunction, including severe injuries caused by sports, acute respiratory distress syndrome (ARDS), pneumonia, COVID‐19, chronic obstructive pulmonary disease, stroke, traumatic brain injury, coma, and anaphylaxis. In addition, for patients under general anesthesia, the use of MV is essential to ensure adequate gas exchange during the procedure [1, 3]. Overall, MV plays a crucial role in both the intensive care settings (ICU) and the operating environments. However, it contributes to ventilator‐induced lung injury (VILI) in 15%–50% of cases, significantly increasing morbidity, mortality, and healthcare costs worldwide.

**FIGURE 1:**
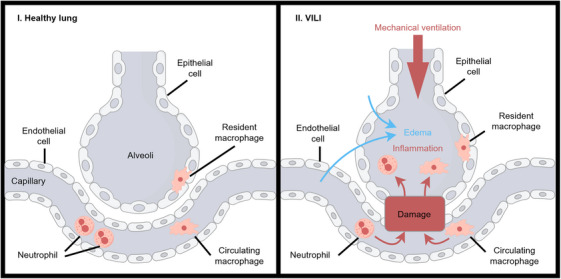
The schematic diagram of healthy lung and injured lung after MV. In the healthy lung, ECs and epithelial cells construct an intact alveolar‐capillary barrier that conducts gas exchange between the blood and the alveolar space. In the progress of VILI, MV disrupts the alveolar‐capillary barrier and promotes the infiltration of circulating *inflammatory* cells into the alveolar space. Activated *inflammatory* cells release various pro‐inflammatory cytokines to induce inflammation. Then, the increase in alveolar osmotic pressure causes fluid from plasma and interstitial fluid to leak into the alveolar space, causing pulmonary edema. The figure was created by Figdraw (www.figdraw.com).

MV delivers oxygenation and carbon dioxide removal when spontaneous breathing is insufficient. Although MV plays a vital role, it also carries risks, including VILI, diaphragmatic atrophy, and impaired hemodynamics [4, 5]. The features of VILI include barotrauma, volutrauma, atelectrauma, and biotrauma [6]. Ventilation with low tidal volume and appropriate positive end‐expiratory pressure (PEEP) minimizes lung injury and postoperative complications, but the risks of volutrauma and atelectrauma persist at varying ventilation settings [3]. Endothelial cells (ECs) and epithelial cells together form the alveolar‐capillary barrier. Damage to these cells leads to increased vascular leak, resulting in the influx of proteins into the alveoli and the infiltration of leukocytes during VILI [7, 8]. Immune cells, primarily neutrophils and macrophages, play a crucial role in the activation, transduction, and amplification of inflammatory signaling that contributes to VILI [8] (Figure 1).

Studies including our own have revealed that abnormal mechanical forces are key factor in the progression of VILI and cardiovascular diseases [9, 10, 11, 12, 13, 14, 15, 16]. However, the exploration of the mechanical mechanisms is still incomplete, and a huge gap exists between these mechanisms and clinical translation. Furthermore, lung injury caused by MV exhibits significant individual differences, and the development of personalized treatment is far behind schedule.

VILI poses a significant challenge in critical care, resulting from mechanical forces during life‐support ventilation. This review summarizes the types of mechanical stress and classical injury patterns in VILI, discusses the mechanotransduction machinery in the lung, and dissects cell‐specific responses in endothelial, epithelial, and immune cells, along with cellular crosstalk in VILI. Current therapeutic approaches include protective ventilation, pharmacological interventions, mechanotransduction inhibitors, and regenerative therapies, though limitations persist in preclinical models and clinical translation. Future directions focus on AI‐enhanced biomechanics, multi‐omics integration, and personalized ventilation strategies to translate mechanobiology insights into clinical applications.

## 2 Mechanical Stress Induced Mechanotransduction in VILI

### 2.1 Mechanical Forces and Classical Injury Patterns in VILI

Mechanical forces during MV include pressure, cyclic stretch, and shear stress [6]. Pressure is derived from air inflow of ventilator and inspiratory effort of patients, building up transpulmonary pressure (airway pressure minus pleural pressure), which provides a force to inflate the lung. Cyclic stretch is caused by breathing movements transmitted from the pleura/chest wall throughout the respiratory system, whereby the lungs expand and relax, leading to repeated stretching and recoil of alveolar and capillary walls. Shear stress occurs with reopening of atelectatic lung units that develop during MV, as airway collapse or fluid obstruction impedes the bubble progression that is required to reopen the distal airway, with the bubble edge applying shear to the epithelial cells [17]. These mechanical stimuli promote VILI.

The classical injury patterns of VILI include barotrauma, volutrauma, atelectrauma, and biotrauma [6]. The first three injury patterns are directly induced by mechanical forces. Among them, barotrauma results from high transpulmonary pressure; volutrauma results from lung overdistension induced excessive stretch [6]; and atelectrauma results from reopening of atelectatic lung induced high shear stress [6]. Moreover, biotrauma refers to the activation and release of inflammatory cytokines triggered by mechanical injuries, which induces inflammation spreads and injury exacerbation [6].

### 2.2 Tissue‐Level Consequences of Mechanical Stress

Mechanical stress related injury patterns eventually induce structural and functional lesion. Excessive transpulmonary pressure and overdistension damage the dense alveolar‐capillary barrier, increasing the barrier permeability. Consequently edema, hemorrhage and alveolar collapse further impair pulmonary gas exchange [18]. Importantly, inflammatory response arises from mechanical injured tissue, extending injury to pulmonary regions with no obvious mechanical injury through inflammatory cascade [6].

It is well‐established that biotrauma (inflammation) is a critical injury pattern in VILI [6]. Mechanical stress is considered as the most upstream triggering factor of inflammtion in pulmonary cells including ECs, epithelial cells, and various immune cells, as these cells initially respond to mechanical stimuli by mechanosensitive receptors [19]. Inflammatory cascade extends this harmful response to entire lung, and inflammatory cytokines are further delivered to the whole body by the circulatory system, inducing systemic inflammation [20]. Multiple organ dysfunction such as heart [21], kidney [22], gut [23], and brain [24] results from MV induced systemic inflammation is always clinical challenge.

### 2.3 Mechanical Sensing Structures and Intracellular Signaling Pathways

In humans, lung consists of less than 10% real tissue, about 10% blood, and more than 80% air [25]. The volume of ductal and alveolar airspaces changes with respiratory cycle, leading to a deformation of alveolar walls and fiber systems [25]. In this process, alveolar epithelial cells are directly stretched due to their connection with the basal lamina [25]. Moreover, mechanical forces acting on extracellular matrix (ECM) are transmitted to the mechanosensitive elements of cells through cell‐matrix and cell–cell junctions [25].

Pulmonary intracellular mechanotransduction signaling pathways have been detailed reviewed by Burgess JK and Gosens R [26]. In short, there are several aspects for pulmonary cells to sense mechanical stress including integrin, cytoskeleton, and bronchoconstriction, as well as afferent nerves and ion channels [26]. Integrin on cell surface directly senses the mechanical changes of ECM and triggers intracellular mechanotransduction, such as classical Src/focal adhesion kinase (FAK) signaling [26]. Src/FAK signaling further activates other downstream pathways such as p130Cas and Hippo‐Yes‐associated protein (YAP)/TAZ [26]. Integrin related signaling regulates the contraction and rearrangement of cytoskeleton, and bronchoconstriction can also activate intracellular signaling such as epidermal growth factor [26]. In addition, nerve fibers sense mechanical stress and initiate intracellular mechanotransduction through ion channels such as TRP channels and Piezo channels [26].

### 2.4 Organelle‐Level Mechanotransduction

Cytoskeleton and ion signaling are critical for intracellular mechanotransduction, which transform macroscopic mechanical stress to microscopic biochemical signals. Further, biochemical signals transmit between intracellular components, regulate cellular organelle functions, ultimately influence phenotype.

There are sufficient evidence indicates that organelle‐level mechanotransduction broadly regulates functions and phenotypes physiologically and pathologically. ECM stiffening promotes microtubule glutamylation to enhance its stabilization, which results from mechano‐dependent glutamine metabolism reprogramming [27]. The features of actomyosin cytoskeleton can regulate cellular sensitivity to mechanical stress, thereby to regulate glycolysis [28]. These mechano‐induced metabolism reprogramming continuously regulates cellular proliferation, migration, differentiation, and apoptosis [27, 28]. Mechanical stress from normal respiration regulate cell division cycle 42 and Protein Tyrosine Kinase 2 expression through integrin/FAK signaling to modulate actin cytoskeleton arrangement, further influence nuclear lamina–chromatin interactions to maintain alveolar cell phenotype [29]. Furthermore, mechanotransduction is critical for intracellular Ca^2+^ homeostasis to protect normal functions of mitochondria and endoplasmic reticulum [30, 31].

## 3 Cell‐Specific Mechanisms in VILI

### 3.1 Capillary ECs

**FIGURE 2:**
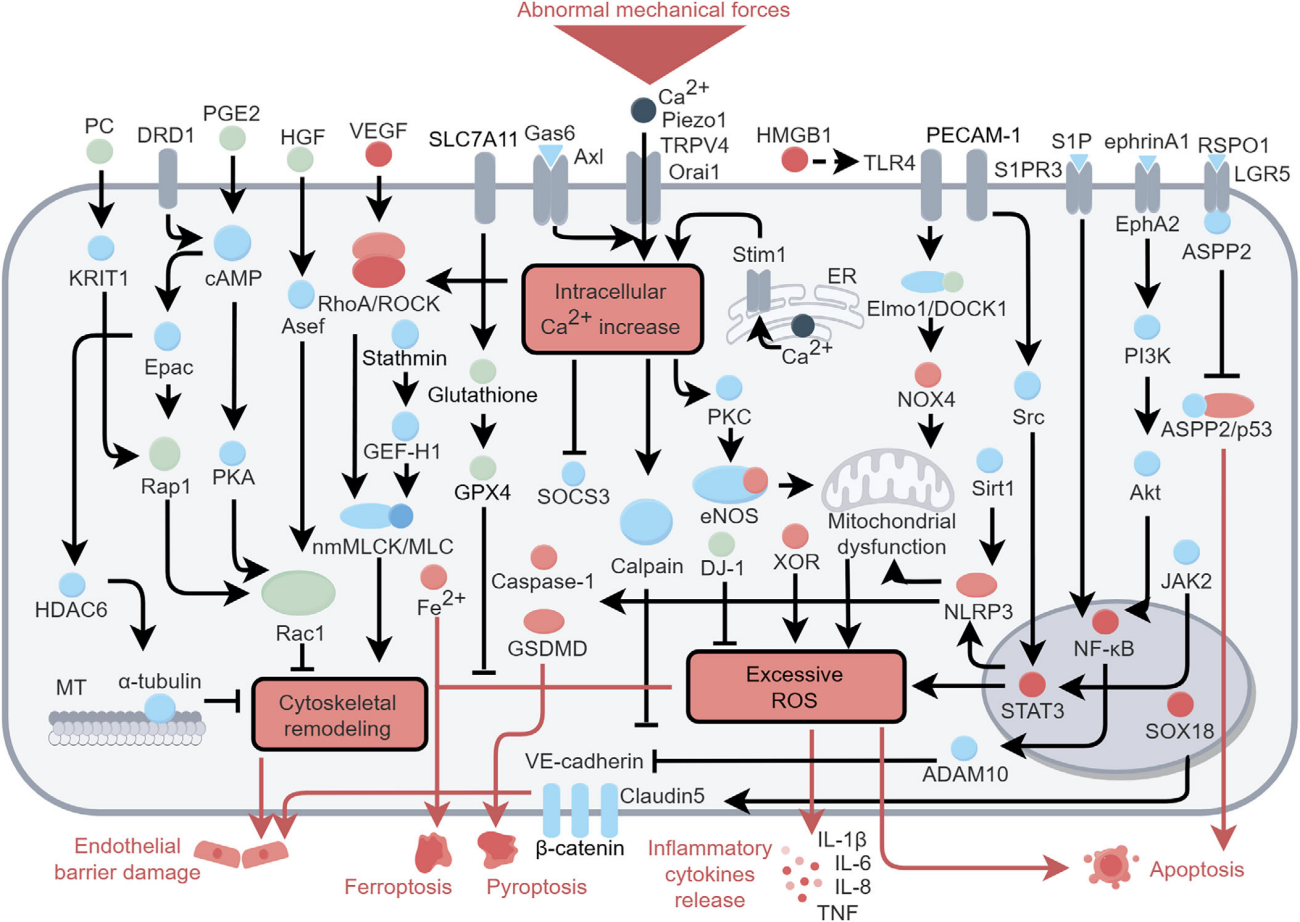
Activated signal pathways in capillary ECs during VILI. Abnormal mechanical forces generated by MV activate mechanosensitive ion channels, while circulating molecules influence ECs within the mechanical microenvironment. Three main responses occur in ECs, including Ca^2+^ elevation, cytoskeletal remodeling, and excessive ROS production. Increased Ca^2+^ is an upstream regulator of cytoskeletal remodeling and ROS production, while Ras superfamily regulates cytoskeletal dynamics and excessive ROS is dependent on mitochondrial dysfunctions. Subsequently, EC barrier damage, inflammatory cytokine release, and apoptosis lead to pulmonary microvascular endothelial injury. The figure was created by Figdraw (www.figdraw.com).

In mammalian lungs, alveoli and capillaries form a continuous, tight barrier to facilitate gas exchange [32]. MV with inappropriate parameters directly causes mechanical damage to ECs [7]. Main signaling networks in ECs during VILI are shown in Figure 2.

#### 3.1.1 Mechanotransduction

Continuous advances in mechanobiology are providing candidate mechanisms for VILI. For, example, TRPV4 activation appears to be harmful during VILI in isolated lungs ventilated at frequencies (40 min^−1^) low for mouse [33]. Inhaled TRPV family inhibitor ruthenium red blocked increased permeability and pulmonary edema in VILI in an ex vivo mice [34]. In high‐fat diet mice, the inhibition of TRPV4/Ca^2+^ signaling pathway by adipose‐derived stem cell attenuated MV‐induced changes in cell–cell junction proteins, β‐catenin and vascular endothelial (VE)‐cadherin and the proinflammatory cytokines interleukin (IL)‐6 and tumor necrosis factor‐α (TNF‐α) [35]. The phosphorylation of eNOS at T495 by Protein Kinase C (PKC) promoted eNOS uncoupling leading to increased production of reactive oxygen species (ROS) and mitochondrial redistribution and dysfunction [36].

Endothelium‐specific knockout of the large mechanosensitive protein Piezo1 in mice enhances endothelial barrier injury in response to high tidal volume (HTV) MV [37]. In contrast, Piezo1 downregulation by shRNA or GSMTx4 (a non‐selective cationic mechanosensitive channel inhibitor) in rat attenuates VILI [38, 39]. These contradictory effects of Piezo1 manipulation may be influenced by: (1) The duration of MV, as 30 min of cyclic stretch on ECs stabilizes membrane VE‐cadherin [37], whereas 4 h ventilation promotes its internalization [38]. (2) The activation of different downstream effectors, as protective Piezo1 signaling in mice involves Src activation [37], whereas detrimental effects in rats are associated with RhoA/ROCK pathway activation [39]. (3) Methodological differences in experimental perturbation, such as the use of an endothelium‐specific knockout model in mice compared to broad‐spectrum shRNA‐mediated knockdown in rats, may reflect the net result of both exacerbatory and protective effects of Piezo1 across multiple cell types [37, 38]. Desensitization of Axl, a tyrosine kinase receptor, to its ligand growth arrest‐specific 6 (Gas6) is linked to Ca^2+^ influx through stretch‐activated calcium channels (including TRP channels and Piezo) [40]. This Ca^2+^ influx leads to a decrease in the levels of the anti‐inflammatory suppressor of cytokine signaling 3 (SOCS3) [41]. Mobilization of cytoplasmic Ca^2+^ from the endoplasmic reticulum calcium stores also contributes to calcium overload, endothelial hyperpermeability, and lung injury in VILI. Stromal‐interacting molecule 1 (Stim1) is involved in Ca^2+^ release from the endoplasmic reticulum and in calcium release‐activated calcium modulator 1 (Orai1) activated PKC [42].

Integrins are transmembrane heterodimeric receptors comprising α and β subunits that transfer mechanical signals between ECM and cytoskeleton [43]. Integrin αvβ5 mediates activation of ECM latent transforming growth factor‐β (TGF‐β) to increase pulmonary vascular leakage by activation of the actin cytoskeleton, as evidenced by β5 subunit knockout mice being protected against lung vascular injury after HTV MV [44].

#### 3.1.2 eNOS and No Production

Physiological levels of nitric oxide (NO) contribute to bronchodilation, surfactant production, mucous secretion, and prevention of inflammation [45]. EC‐specific overexpression of eNOS in mice inhibits VILI with reduced production of inflammatory cytokines, neutrophil infiltration, and lung edema [46]. The peptide Ac‐ANX‐A1 FPR2 agonist cleaved from the N‐terminal of annexin A1 promotes phosphorylation of eNOS through to Akt activity thereby reducing endothelial injury during HTV MV [47]. However, eNOS knockout mice subjected to HTV MV have less injury than wild type mice. This improvement is attributed to a reduction in superoxide levels resulting from decreased uncoupled eNOS activity [48], whereas normally eNOS‐derived NO functions as a vasculo‐protective factor. Uncoupling occurs in the context of reduced availability of L‐Arginine. Surprisingly, L‐arginine supplementation does not appear to have been evaluated in VILI. L‐Arginine has no impact on preterm newborn ARDS oxygenation [49], and in adult sepsis increases NO production without improving tissue perfusion [50].

#### 3.1.3 Ras Superfamily in Cytoskeletal Dynamics

Ras superfamily of small GTPases regulate GDP/GTP transformation [51]. RhoA, Rap1, and Rac1 have been found to play crucial roles in the pathological processes of VILI due to their impact on the cytoskeleton and the integrity of intercellular junctions.

Activated RhoA/ROCK leads to the phosphorylation of myosin light chain in ECs which induces cytoskeletal reorganization and contributes to vascular leakage in rats subjected to HTV MV [52]. The two‐hit model, which simulates the combined pathophysiological effects of sepsis and MV, revealed a significant upregulation of sphingosine kinase 1 [53]. This leads to the activation of RhoA/ROCK and endothelial hyperpermeability [53]. Y‐27632, a selective ROCK inhibitor, significantly attenuated inflammatory responses induced by HTV MV in mice [54]. These findings underscore the critical involvement of Rho/ROCK signaling pathway in mediating pulmonary inflammatory responses during MV and concurrent infection [54]. Stathmin destabilizes microtubules releasing microtubule‐bound Rho‐specific guanine nucleotide exchange factor H1 (GEF‐H1), thereby activating Rho and facilitating cytoskeletal remodeling [55, 56]. Knockdown of stathmin or GEF‐H1 protects endothelial barrier function in VILI [56, 57].

In contrast to the detrimental effects of RhoA/ROCK pathway, both Rac1 and Rap1 play a protective role in maintaining endothelial barrier. Hepatocyte growth factor (HGF), which shows elevated levels in patients with ventilated ARDS [58], enhances endothelial barrier integrity by activating Rac1‐GTPase and p21‐activated kinase 1. This mechanism counteracts the increase in endothelial permeability induced by vascular endothelial growth factor (VEGF) and Rho‐GTPase signaling [59]. HGF‐induced Rac1 activation is partly depended on Asef, a Rac1‐specific GEF, which facilitates peripheral cytoskeletal remodeling and the formation of adherent junctions [60].

Prostaglandin E_2_ (PGE_2_) also mitigates endothelial barrier dysfunction during VILI partly through its effects on cyclic AMP (cAMP) [61, 62]. Increased cAMP activates PKA and PKA‐independent Epac (a Rap‐specific GEF)/Rap1 to induce Rac1 activation [59, 60]. Activation of dopamine D1 receptor leads to the stimulation of the cAMP/Epac pathway, which reduces α‐tubulin deacetylation by inhibiting histone deacetylase 6 [63]. This process enhances microtubule stability and strengthens the EC barrier [63]. In addition, prostacyclin induces Rap1 to recruit its effector, Krev interaction trapped‐1, to *adherens* junctions, thereby maintaining the integrity of the EC monolayer [64, 65]. This mechanism also accounts for the protective effects of Rap1 under conditions of high cyclic stretch and HTV MV [64, 65].

#### 3.1.4 Oxidative Stress and Inflammation

ROS are byproducts of cellular metabolism and exhibit high oxidative activity. NADPH oxidase (NOX), eNOS, mitochondria, and xanthine oxidase are the major enzymatic sources of ROS in the circulatory system [66, 67]. Excessive or pathological levels of ROS induce oxidative stress, inflammatory disorders, and circulatory system diseases [66, 68].

After MV‐induced tissue damage, damage‐associated molecular patterns (DAMPs) are released into the extracellular space, which is considered to mark the beginning of biotrauma [69]. High‐mobility box group‐1 (HMGB1), one of DAMPs, is elevated in both HTV MV mice and ECs subjected to high cyclic stretch [70]. Extracellular HMGB1 induces mitochondrial dysfunction, the loss of adherent junctions and vascular leakage [70]. Another DAMP protein, extracellular nicotinamide phosphoribosyl transferase (eNAMPT), activates TLR4 that reduces DOCK1 (a Rac1‐specific GEF) and its binding protein Elmo1 [71]. Decreased DOCK1 not only promotes NOX4‐related ROS production but also inhibits non‐muscular myosin light‐chain kinase (nmMLCK)/MLC‐regulated endothelial barrier‐enhancing lamellipodia formation [71]. However, global knockout of nmMLCK protects against lung injury in mice challenged with lipopolysaccharide (LPS)/VILI. These findings suggest that nmMLCK exerts distinct effects on inflammation and EC barrier function [71, 72]. Moreover, the reduction of sirtuin 1 (Sirt1) levels activates the NOD‐like receptor thermal protein domain associated protein 3 (NLRP3) inflammasome, which partially contributes to mitochondrial damage and increased vascular leakage in VILI [73]. Under LPS‐induced acute lung injury conditions, RhoA/ROCK‐promoted eNOS uncoupling is an important mechanism underlying excessive ROS production and NLRP3 activation [74]. In turn, eNOS uncoupling contributes to RhoA activation (via nitration at Tyr34), increasing vascular leakage and inflammation [75]. In vascular cells, it is well‐established that RhoA/ROCK and eNOS/NO are mutually antagonistic [76]. Specifically, ROCK decreases eNOS expression by destabilizing eNOS mRNA [77]. Conversely, NO can activate Protein Kinase G (PKG), which subsequently phosphorylates RhoA to inhibit its translocation to membrane, thereby preventing RhoA activation [78]. However, it remains unclear as to whether a similar mechanism exists in VILI. PECAM‐1‐mediatied activation of the Src/signal transducer and activator of transcription (STAT3) pathway promotes NLRP3 inflammasome assembly, leading to caspase‐1 activation, cleavage of Gasdermin‐D (GSDMD), and subsequent EC pyroptosis [79, 80]. HTV MV also elevates the activity of xanthine oxidoreductase (XOR), which has the capability to generate ROS, and activates ERK and p38 mitogen‐activated protein kinase (MAPK) pathways [81].

Erythropoietin‐producing hepatoma receptor tyrosine kinase A2 activates the phosphoinositide 3‐kinase/Akt/NF‐κB pathways and contributes to the inflammatory response in VILI [82]. Activation of Janus kinase 2 (JAK2)/STAT3 pathway promotes EC ROS production and apoptosis in VILI [83]. Parkinson's disease protein 7 (DJ‐1 or PARK7) attenuates oxidative stress and cell death in LPS/VILI mice [84]. In vitro, pathogenic stretch leads to increased lipid peroxidation, ROS production, and ferrous ion (Fe^2+^) accumulation, along with decreased solute carrier family 7 member 11 (SLC7A11) which is required for cystine/glutathione homeostasis and glutathione peroxidase 4 (GPX4). These pro‐oxidative changes indicate that ferroptosis may represent an alternative cell fate in VILI [85].

#### 3.1.5 Other Molecular Mechanisms

Beyond the aforementioned mechanisms, emerging evidence suggests that additional molecular pathways contribute to mechanical stress‐induced pulmonary capillary endothelial injury. HTV MV damages endothelial intercellular junctions through a lipid metabolism‐related pathway, in which the sphingosine‐1‐phosphate (S1P)/S1P receptor 3/NF‐κB pathway activates ADAM10 (a metalloprotease), leading to the cleavage of VE‐cadherin [86]. Loss of transcription factor SOX18 inhibits Claudin‐5 expression and induces endothelial barrier damage in VILI [87]. Xu et al. elucidated that MV suppresses R‐spondin1 expression, which normally promotes leucine‐rich repeat containing G‐protein coupled receptor 5‐ apoptosis‐stimulating protein of p53 2 binding to inhibit p53‐driven apoptosis in mechanically stressed lung ECs. Restoring R‐spondin1 signaling could thus represent a novel strategy to reduce VILI [88]. Consequently, mechanical stress‐induced pulmonary endothelial injury emerges as a pivotal therapeutic target in mitigating ventilator‐associated lung damage.

#### 3.1.6 Summary and Future Remarks

In VILI, studies on lung ECs have primarily focused on capillaries rather than large vessels, mainly due to the critical effects of capillary barrier damage on inflammatory diffusion and impairing pulmonary function. Endothelial barrier leakage arises from cytoskeletal remodeling, loss of cellular junction proteins and cell death, which collectively promote neutrophil infiltration and increased levels of inflammatory cytokines. In this progressive condition, mechanical stress acts as an initial trigger, after which the mechanical signals are transmitted intracellularly by biochemical signaling. ROS production, cell death, and inflammation are not merely terminal pathological phenomena, but also act as key positive feedback drivers of deterioration due to cascade reactions. However, how the network of inflammatory cytokines further regulates endothelial signaling remains poorly understood. Additional contributing factors‐such as dysregulated microvascular hemodynamics and the presence of blood‐borne mediators, including circulating DAMPs, extracellular vesicles (e.g., microparticles), and activated platelets‐should also be considered.

### 3.2 Alveolar Epithelial Cells

**FIGURE 3:**
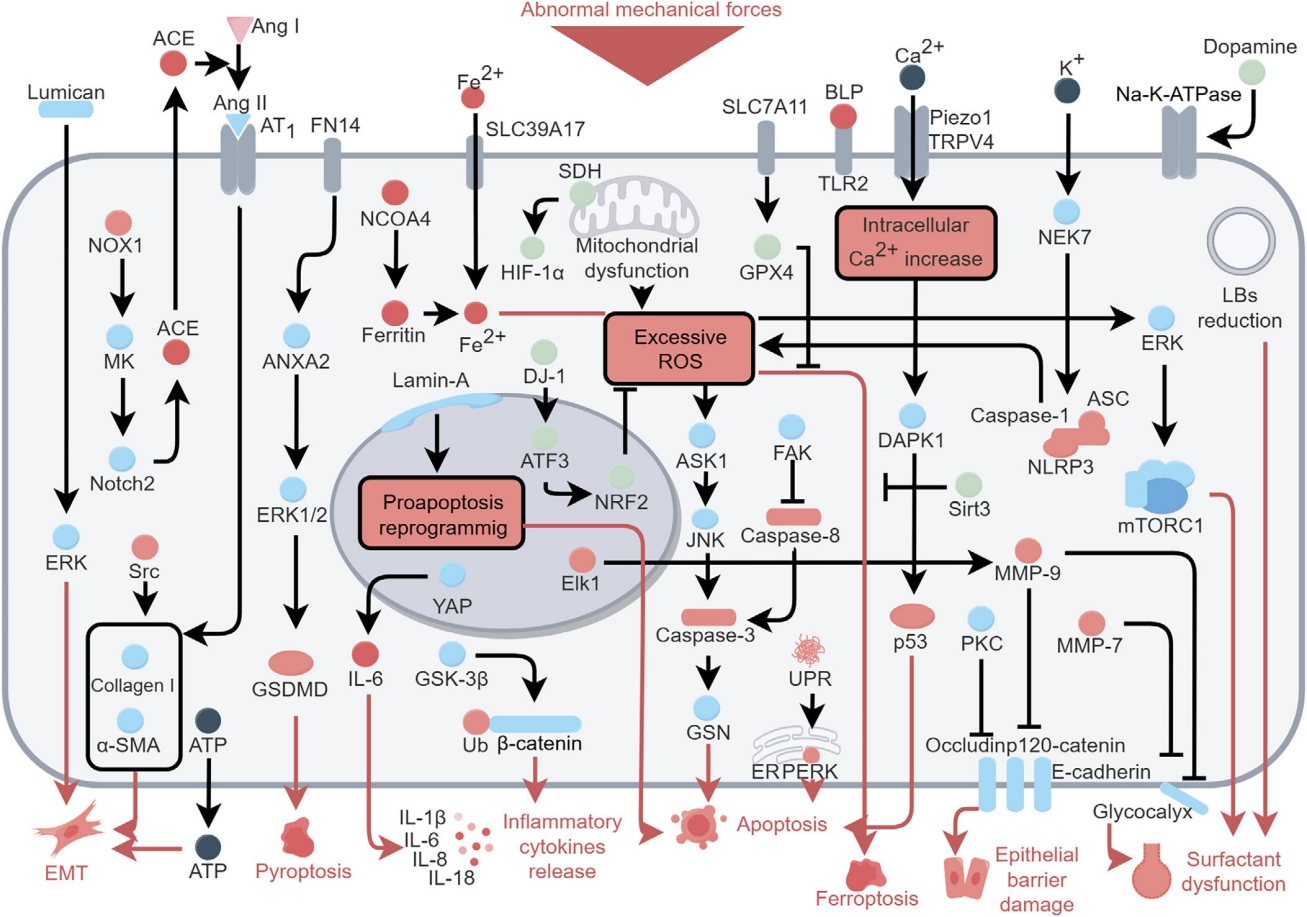
Mechanical stress induced signal transduction in alveolar epithelial cells during VILI. Abnormal mechanical signaling in alveolar epithelial cells (both ATI and ATII) triggers cytoplasmic Ca^2+^ elevation, ROS production, and proapoptotic reprogramming. Epithelial cell apoptosis and inflammatory cytokines release are main reasons for epithelium injury and local inflammation. EMT contributes to the development of lung fibrosis with the progress of pathology. The impaired ATII signaling induces surfactant dysfunction. The figure was created by Figdraw (www.figdraw.com).

Similar to ECs, alveolar epithelial cells exhibit mechanotransduction pathways, while also having distinct roles in barrier repair and surfactant homeostasis. The alveolar epithelium is composed of approximately 95% alveolar type I (ATI) cells, which adhere to capillaries and form a tight alveolar‐capillary barrier, and about 5% alveolar type II (ATII) cells, which secrete surfactant and play a role in self‐renewal or generation of ATI cells during lung repair [32, 89]. The major signaling pathways of alveolar epithelial cells during VILI are shown in Figure 3.

#### 3.2.1 Mechanotransduction

Both TRPV4 and Piezo1 in epithelial cells sense harmful mechanical stimuli [90, 91]. In vitro, mechanical stretch‐induced Ca^2+^ influx is dependent on TRPV4, leading to increased release of IL‐6 and IL‐8 [90]. Epithelial cell‐specific knockout of Piezo1 in mice attenuates MV‐induced injury, similar to the effects observed in ECs [37]. In addition, the deletion of epithelial Piezo1 diminishes EMT‐related pulmonary fibrosis by inhibiting calcium‐induced extracellular ATP release [91]. HTV MV‐induced Ca^2+^ signaling also activate death‐associated protein kinase 1 (DAPK1), thereby promoting p53‐related apoptosis and epithelial injury [92].

Moreover, intracellular mechanoresponsive signaling pathways were also explored. Increased expression of mTOR Complex I (mTORC1) is found in mice subjected to HTV MV injury and in patients on ventilators [93]. The activation of mTORC1 induced by ROS/ERK during VILI is associated with surfactant dysfunction [93]. This dysfunction may be due to impaired release of surfactant secretagogue ATP from ATI to ATII, leading to alveolar collapse and decreased lung compliance [93]. In vitro study demonstrates that YAP pathway promotes IL‐6 expression in rat ATII cells in response to high levels of cyclic stretch [94]. The transduction of mechanical stress ultimately reaches the nucleus, which may be important for epigenetic regulation of inflammatory and fibrogenic cytokine expression. MV induced Lamin A elevation is associated to activated proapoptotic genes [95]. Zmpste24 knockout mice (with inhibited Lamin A maturation) exhibit lower lung injury scores and less leakage after ventilation injury [95].

#### 3.2.2 Oxidative Stress and Inflammation

Consistent with findings in ECs, oxidative stress, and inflammatory signaling are activated in alveolar epithelial cells during MV [94, 96, 97, 98]. In mice with VILI, elevated ROS impairment epithelial cell function is partially mediated by the redox‐sensitive Apoptosis Signal‐regulating Kinase‐1 (ASK‐1) pathway [96, 97]. The induction of ASK‐1 leads to the activation of c‐Jun NH2‐terminal kinases (JNK), which subsequently activates caspase‐3 [96, 97]. Caspase‐3 is a main executioner effector that cleaves various substrates to promote apoptosis [99] and gelsolin (GSN) is one of substrates increased in the lungs of VILI mice, especially in ECs and epithelial cells [100]. Knockout of GSN in mice prevents apoptosis and reduces inflammatory cytokine secretion after HTV MV [100].

Different apoptosis‐related pathways are found in ATI and ATII. In ATI, abnormal mechanical forces generated by MVs trigger a sustained unfolded protein response (UPR) and the protein kinase RNA‐like endoplasmic reticulum kinase (PERK, a UPR sensor), subsequently promoting IL‐18 secretion and apoptosis [101, 102]. In ATII, inhibition of GSK3β pathway ameliorates MV‐induced inflammation and apoptosis in association with restoration of WNT/β‐catenin levels [98]. Notably, these pathways have not been shown to be unique in ATI or ATII, and cell‐specific mechanisms require further exploration.

Epithelial cells contribute to inflammation through NLRP3 activation [103]. This process involves the assembly of NLRP3 with apoptosis‐associated speck‐like protein (ASC) and caspase‐1, leading to the formation of the NLRP3 inflammasome [103]. In VILI mice, activated NIMA‐related kinase 7 (NEK7) is necessary to NLPR3 inflammasome formation, which increases IL‐1β secretion and the degradation of cell junction proteins (p120‐catenin and occludin) [104, 105]. MV triggered intracellular K^+^ efflux may be critical to NEK7 activation [105]. Indeed, blockade of IL‐1 receptor protects against epithelium injury in HTV MV rats [106]. Moreover, epithelial cells subjected to cyclic stretch exhibit upregulated TLR2, which heightens their sensitivity to bacterial lipopeptides (BLP) [107].

DJ‐1 is increased in epithelial cells to resist oxidative stress and inflammation through protecting against proteasomal degradation of nuclear factor erythroid 2‐related factor 2 (NRF2), a transcription factor that coordinates numerous antioxidant genes expression [84, 108]. Activating transcription factor 3 is the upstream regulator of DJ‐1 that alleviates inflammation and decreases epithelial cells permeability [108]. Targeting alveolar‐epithelial succinate dehydrogenase dampens VILI via succinate‐mediated stabilization of hypoxia inducible factor‐1 α [109, 110].

In epithelial cells, pathogenic cyclic stretch activates FN14/ANXA2/ERK1/2 axis to promote the transcription of GSDMD, IL‐1β, and IL‐18, thereby inducing pyroptosis and inflammatory responses [111]. Pathogenic stretch also induces a ferroptosis response similar to that occurring in ECs, including lipid peroxidation, ROS production, and decreased expression of GPX4 and SLC7A11. Fe^2+^ accumulation is partly contributed by the upregulation of solute carrier family 39 member 17 (SLC39A17), an ion transport protein [85], and increased levels of NCOA4 that mediates ferritin degradation [112]. Recently, Lin et al. demonstrated that capsaicin attenuates ferroptosis in VILI by enhancing SIRT3 activity, thereby mitigating mitochondrial oxidative damage and preserving redox homeostasis. These findings highlight capsaicin's TRP receptor as a novel potential therapeutic target for VILI [113].

#### 3.2.3 Epithelial–Mesenchymal Transition

Epithelial–mesenchymal transition (EMT) is a differentiation process in which epithelial cells lose epithelial marker (E‐cadherin) and transform into (myo)fibroblast phenotype (acquire mesenchymal markers α‐smooth muscle actin, α‐SMA), which contributes to lung fibrosis [114]. HTV MV promotion of EMT is evidenced in both in vitro and in vivo settings [115].

Over half of ARDS patients with MV suffer from lung fibrosis [116]. Under HTV MV, EMT is implicated in the fibroproliferative phase that follows initial inflammatory response and epithelium injury, revealing an epithelial‐specific mechanism of VILI [117]. These findings align well with recent advances in understanding of the origins of other forms of pulmonary fibrosis in interstitial lung disease which recognize the importance of epithelial injury [118]. Proto‐oncogene tyrosine‐protein kinase Src is critical to HTV MV induced EMT as Src‐deficient mice express lower fibrogenic markers (α‐SMA and collagen I) with reduced inflammation and apoptosis [117, 119]. However, FAK of epithelial cells sequesters caspase‐8 to prevent apoptosis and fibrosis although activated FAK recruit and further activate Src [120]. In HTV MV mice, downregulation of SRY‐box transcription factor 11 (SOX11) induced FAK suppression, suggesting a potential role of SOX11 in ventilator‐related lung fibrosis [121].

Renin‐angiotensin system is important in EMT under HTV MV. Angiotensin‐converting enzyme (ACE) converts angiotensin (Ang) I to Ang II, then Ang II activates Ang II type 1 (AT_1_) receptor to increase α‐SMA expression and collagen deposition [122, 123]. The heparin‐binding growth factor midkine (MK) is related to EMT as circulating MK increase in ventilated ARDS patients, and MK knockout alleviates ventilator‐induced lung fibrosis in mice. Mechanical stretch activated Nox1/MK/Notch2/ACE pathway is also found in epithelial cells [124]. Lumican, one of the proteoglycans that constitutes pulmonary ECM, activates ERK1/2 signaling to promote EMT and lung fibrosis in HTV MV mice [125]. In addition, autotaxin secreted by bronchial epithelial cells contributes to VILI pathogenesis including its complication of lung fibrosis in survivors of MV [126, 127].

#### 3.2.4 Lung Liquid Clearance and Surfactant Secretion

Alveolar epithelial cells play crucial roles in maintaining lung fluid homeostasis. These cells absorb sodium (Na^+^) from lung liquid through apical epithelial sodium channel (ENaC) and extrude Na^+^ through basolateral Na‐K‐ATPases. This process creates an ion concentration gradient to benefit lung liquid clearance (LLC) [128].

LLC is significantly impaired in HTV MV rats, whereas overexpression of Na‐K‐ATPase α2 protein restores LLC [129]. Dopamine increases the activity of Na‐K‐ATPase in ATII, indicating the protective effects of nervous system on LLC [130]. However, the role and mechanisms of ENaC in epithelial cells need further exploration.

Similarly to LLC, surfactant secretion is also a critical process to maintain normal alveolar functions. Surfactant secretion is a specific function of ATII to reduce alveolar surface tension and conserve normal lung compliance [131]. In ATII, several of lipids and surfactant‐related proteins are packaged into lamellar bodies (LBs) to synthesize surfactant, which is then secreted by ATII via exocytosis [132]. HTV MV decreases the number and area of LBs in rat lung and impaired the ability of LBs to reduce surface tension [132]. Increased matrix metalloproteinase (MMP)‐7 and MMP‐9 promote the degradation of alveolar epithelial glycocalyx (a layer of glycosaminoglycans between epithelium and surfactant), which is an important cause of surfactant dysfunction and micro‐atelectasis in ventilated ARDS patients [133].

#### 3.2.5 Other Molecular Mechanisms

Alveolar epithelial tight junction impairment plays an important role in epithelial hyperpermeability. HTV MV activates PKC to inhibit the expression of occludin in alveolar epithelial cells [134]. HTV MV activates the transcription factor ETS‐domain containing protein, resulting in increased MMP‐9/TIMP‐1 ratio [123]. This shift inhibits the expression of E‐cadherin and occludin, thereby contributing to VILI [135]. HTV MV also increases S1P lyase in lung tissue to promote S1P catabolism, which induces apoptosis, inflammatory cytokines secretion, and paracellular gap formation [136].

#### 3.2.6 Summary and Future Remarks

Alveolar epithelial cells assume barrier function similar to capillary ECs with both cell types collectively form continuous tight alveolar‐capillary barrier, which is the basis of lung gas exchange and microenvironment homeostasis. MV is prone to imposes abnormal mechanical forces on lung tissue, damaging alveolar epithelial cells directly as well as capillary ECs jointly. Possibly due to the thickness of the alveolar‐capillary barrier is less than 1 µm [137], the functions of epithelial cells in VILI are similar to those of ECs. These include mechanosensation, the protective effects of NO [138], oxidative stress, cell death, and inflammation. Furthermore, ECs and epithelial cells respectively activate different signaling pathways underlying similar functions and possess their own specific biological process. In Table 1, we compare the shared and opposing molecular mechanisms and functions of ECs and alveolar epithelial cells in VILI.

**TABLE 1:** Comparison of molecular mechanisms and functions between ECs and alveolar epithelial cells in VILI.

Functional consequence	Comparison		Endothelial cell	Alveolar epithelial cell
Mechanotransduction	Receptors	Common	TRPV4 [35, 36, 90], Piezo1 [37, 38, 39, 91]	
		Different	Axl [40], Stim1 [42]	—
	Effectors	Common	Ca^2+^	
		Different	PKC [36, 42], eNOS [36]	DAPK1 [92], p53 [92]
Oxidative stress	Effectors	Common	NLRP3 [73, 103], DJ‐1 [84]	
		Different	TLR4/DOCK1/Elmo1/Nox4 [71] Sirt1/NLRP3 [73], JAK2/STAT3 [83], XOR [74]	ASK1 [96, 97]/JNK [96, 97]/caspase‐3 [99]/GSN [100], NEK7 [104, 105]/NLRP3/ASC/caspase‐1 [103]
Inflammation	Effectors	Common	IL‐1β [71, 72, 104, 105], IL‐6 [35, 90], IL‐8 [90]	
		Different	TNF [35]	IL‐18 [101, 102]
Apoptosis	Extrinsic Pathways		RSPO1/LGR5/ASPP2/p53 [88]	Piezo1 [91], TRPV4 [90]/Ca^2+^/DAPK1 [92]/p53 [92], caspase‐8/caspase‐3 [119]
	Intrinsic Pathways		JAK2/STAT3/ROS [83]	ASK1 [96, 97]/JNK [96, 97]/caspase‐3 [99]/GSN [100], UPR [101]/PERK [102]
Pyroptosis	Receptors	Different	PECAM‐1 [80]	FN14 [111]
	Effectors	Common	GSDMD [79, 80, 111]	
		Different	Src/STAT3 [80], NLRP3/caspase‐1 [79]	ANXA2/ERK1/2 [111]
Ferroptosis	Receptors	Common	SLC7A11 [85]	
		Different	—	SLC39A17 [85]
	Effectors	Common	Fe^2+^, ROS, GPX4 [85]	
		Different	—	NCOA4/ferritin [112], ACSL4 [85]
Others	Cellular specific functions		eNOS and NO production [46, 47, 48]	EMT [119, 120, 121, 122, 123, 124, 125, 126, 127], LLC [129, 130], surfactant secretion [131, 132, 133]

In the future, common and distinct mechanisms between ECs and epithelial cells need further investigation. For example, ZO‐1 is a component of tight junctions in both EC and epithelial cell membrane. In zebrafish embryo, ZO‐1 senses actomyosin tension to form clusters through phase separation, which is necessary for actin binding and endows ZO‐1 mechanosensitivity [139]. However, the roles of phase separation‐mediated mechanotransduction in VILI remain to be fully elucidated. The regeneration capacity and mechanism of both ECs and alveolar epithelial cells in VILI has also lacked attention. Moreover, it is still unclear how MV regulates the transformation from ATII to ATI, and whether other progenitor cells are involved [140]. MV‐induced hypoxia or hyperoxia may also play important roles [141, 142], as alveolar epithelial cells are directly exposed to the gas phase. Subsequent pathological changes in blood oxygen and carbon dioxide may further impair ECs.

### 3.3 Immune Cells

Immune cells either circulate in the blood or reside in tissues and participate in innate and adaptive responses [143]. In VILI, MV promotes various immune cells recruitment in the lung tissue and amplifies their response to infection, which is the main reason for uncontrolled inflammation.

#### 3.3.1 Neutrophil

**FIGURE 4:**
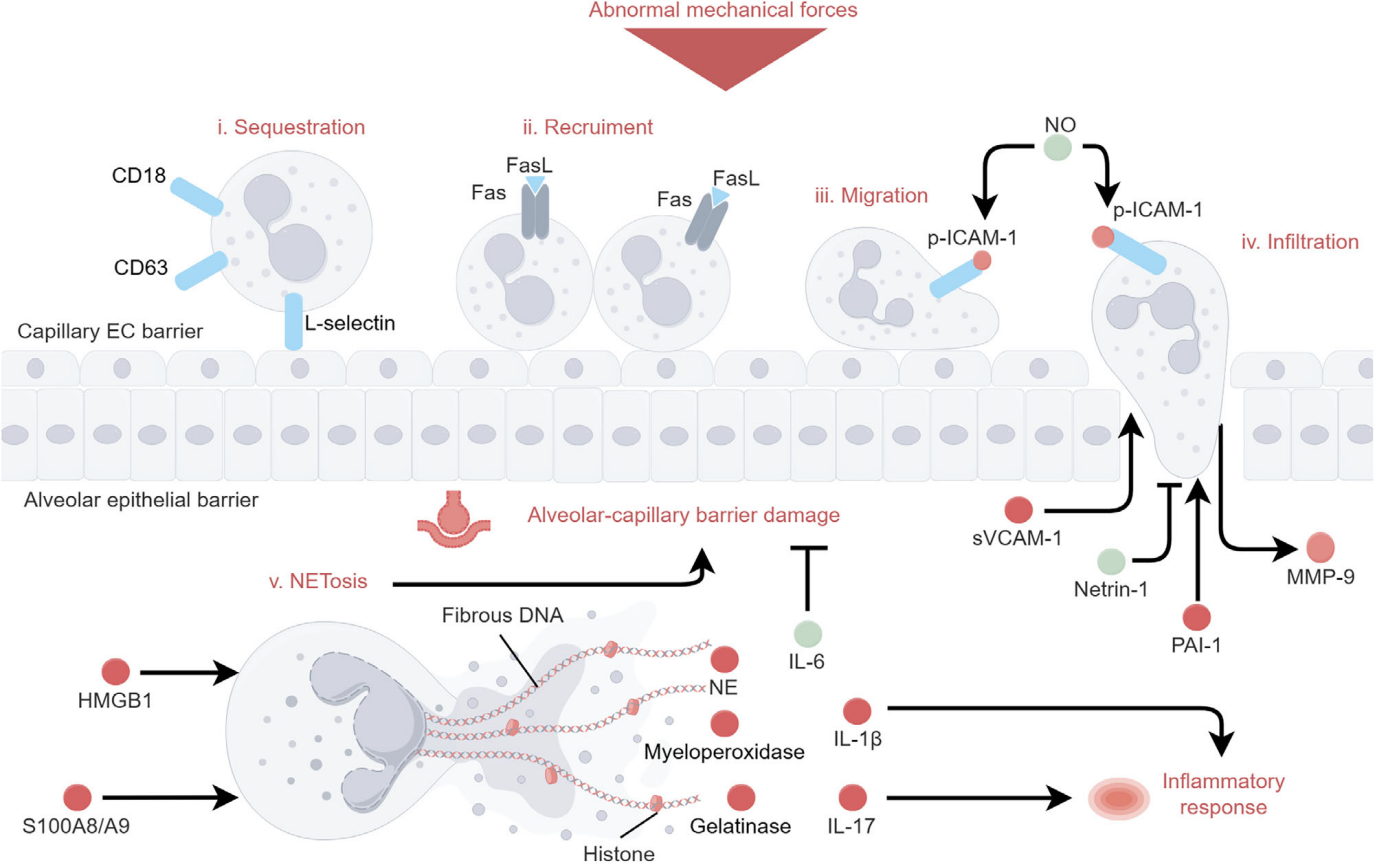
Molecular mechanism in neutrophil during VILI. Under abnormal mechanical stress, neutrophils enter the lung tissue from the circulation system via a series of continuous cascade reactions, including sequestration, recruitment, migration, and infiltration. Then, NETosis occurs in alveolar space, inducing inflammatory response and alveolar‐capillary barrier damage. The figure was created by Figdraw (www.figdraw.com).

Neutrophils play a crucial role in the early inflammatory response and contribute significantly to inflammatory injury during VILI [144, 145]. Neutrophil infiltration is a widespread index to measure alveolar‐capillary leakage and degree of inflammatory response [46, 70, 72, 93, 144]. Specifically, MV triggers neutrophil activation, recruitment, and infiltration, along with the release of neutrophil extracellular traps (NETs) and pro‐inflammatory cytokines. Signaling pathways of neutrophil during VILI are shown in Figure 4.

Neutrophil activation induced by MV has been demonstrated in patients with ventilator‐associated ARDS. This activation is characterized by an increase in oxidant production, elevated adhesion molecules and granule markers such as CD18, L‐selectin, and CD63, and the release of neutrophil elastase (NE) [146]. Early lung neutrophil sequestration is found to depend on L‐selectin rather than CD18 under HTV MV [147]. Fas, a membrane surface receptor that binds to its ligand FasL, is required for neutrophil recruitment [148]. HTV MV‐induced MMP‐9 secretion facilitates neutrophil transmigration, probably due to its degradation of basement membrane [149]. NE is also needed for neutrophil transit across lung barrier in VILI [150]. Moreover, neutrophil infiltration in VILI can be enhanced by various chemoattractants including increased ATI‐derived soluble VCAM‐1 [151], fibrinolytic system‐related plasminogen activator inhibitor type 1 [152] and decreased netrin‐1 [153].

Activated neutrophils engulf and kill bacteria in infected tissue [154]. Meanwhile, NETs, which comprise fibrous DNA, histones and various granule proteins (such as NE, myeloperoxidase and gelatinase), are released into extracellular space [154, 155]. NETs formation (called NETosis) is accompanied by neutrophil death is also found in VILI [156]. MV enhances NETosis, attributing to the increase of DAMPs like HMGB1 and myeloid related protein 8/9 [157, 158]. TLR4 plays a vital role in enabling neutrophils to respond to DAMPs and facilitating the release of NETs [158, 159]. In addition, neutrophil‐secreted IL‐1β and IL‐17 exacerbate inflammation and disrupt the alveolar‐capillary barrier in VILI [160, 161]. However, somewhat counterintuitively IL‐6 secreted by neutrophils plays a protective role for lung barrier by reducing neutrophil adherence [162].

#### 3.3.2 Monocytes and Macrophages

Monocytes are immune effector cells that recognize pathogens, eliminate harmful debris, and release inflammatory cytokines [163]. They are generally classified into Ly6C^low^ and Ly6C^high^[163]. Derived from bone marrow and circulating in the bloodstream, Ly6C^low^ monocytes may transition to alveolar macrophages (AMs) under homeostasis conditions while Ly6C^high^ monocytes are recruited to inflamed tissues and contribute to the pool of inflammatory macrophages [163]. HTV MV promotes the recruitment of Ly6C^high^ monocytes from the lung microvasculature and the formation of pulmonary edema [164]. HTV MV induced Ly6C^high^ recruitment is dependent on cyclooxygenase‐2 expression and partly results from p38 MAPK/MCP‐1 pathway activation [165, 166]. Ly6C^high^ monocytes are an important source of VEGF that increases endothelial leakage in VILI [167].

Although monocytes differentiate into macrophages in a well‐known process that occurs in inflammatory reactions [168], the specific mechanisms in relation to VILI are unclear. Tissue‐resident AMs have garnered more attention in VILI, likely due to their long‐standing presence and their significant response to persistent mechanical stimuli and infections. AMs express CD11c and sialic acid‐binding lectin F instead of CD11b and C‐X3‐C motif chemokine receptor 1 that distinguish them from monocyte‐derived macrophages [169]. Activation of AMs is usually regarded as initial stage of inflammatory cascade and prior depletion of AMs has been shown to effectively prevent VILI [170, 171].

**FIGURE 5:**
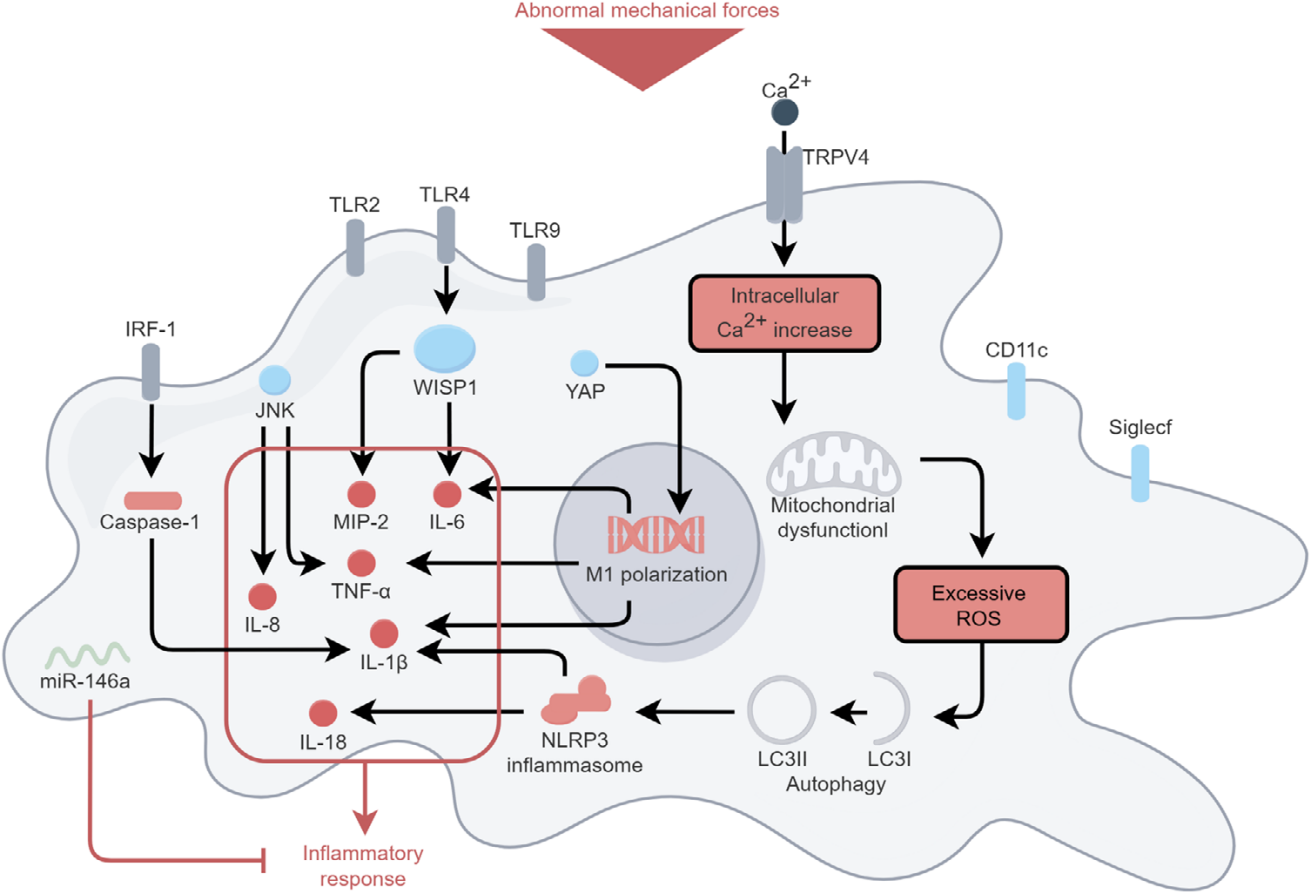
Mechanotransduction in alveolar macrophages during VILI. Under abnormal mechanical stress, macrophages experience increased intracellular Ca^2+^ and excessive ROS. TLR pathways promote the synthesis and release of various pro‐inflammatory cytokines in response to pathogens, cell death pathways, and macrophage polarization, thereby initiating inflammatory responses. The figure was created by Figdraw (www.figdraw.com).

As AMs localize to the luminal surface of alveoli, MV‐induced AM activation also relies on mechanotransduction, including activation of TRPV4 receptor and YAP [172, 173]. AM‐derived miR‐146a expression is mechanosensitive and protects against inflammation, but endogenous elevation of miR‐146a is not sufficient to alleviate lung injury after MV [174]. HTV MV stimulated AMs increase their expression of TLR2, TLR4, and TLR9 that enhance inflammatory signaling [175]. TLR4 promotes AMs to release IL‐6 and MIP‐2 through upregulating WNT1‐inducible signaling pathway protein 1 [176]. HTV MV induced mtROS production in AMs also contributes to proinflammatory cytokine secretion as ROS activate NLRP3 inflammasome [177]. Enhanced autophagy in MV has been linked to the activation of NLRP3 inflammasome and the release of IL‐1β and IL‐18 [177, 178]. Increased IL‐1β from AMs also relies on interferon regulatory factor‐1/caspase‐1 regulated AMs pyroptosis [179]. MV activates JNK pathway in AMs, leading to the release of IL‐8 and TNF‐α [180]. Signaling pathways of AM during VILI are shown in Figure 5.

#### 3.3.3 Summary and Future Remarks

Macrophages and neutrophils are the two major inflammatory cell types that trigger and amplify inflammation in VILI. MV significantly enhances their activation, leading to excessive proinflammatory cytokine levels and subsequent biotrauma. Although both enhance inflammation, neutrophil activation typically occurs earlier than that of macrophages. The inflammatory factors released from these cells exhibit both commonalities and distinct characteristics. Together with ECs and epithelial cells, neutrophils and macrophages drive the inflammatory cascade and induce an inflammatory storm in VILI. While depleting either neutrophil or macrophage populations alleviates VILI in animal models, clinical translation requires deep caution, particularly since many ARDS patients have concurrent infections. Therefore, an ideal therapeutic strategy would inhibit excessive inflammatory cytokine levels and activity without completely compromising innate host defense functions, albeit achieving this balance remains challenging. In addition, immune cells such as invariant natural killer T cells [181] and regulatory T cells [182] are also involved in VILI, but further investigations are needed to better understand their roles.

## 4 Cellular Crosstalk

**FIGURE 6:**
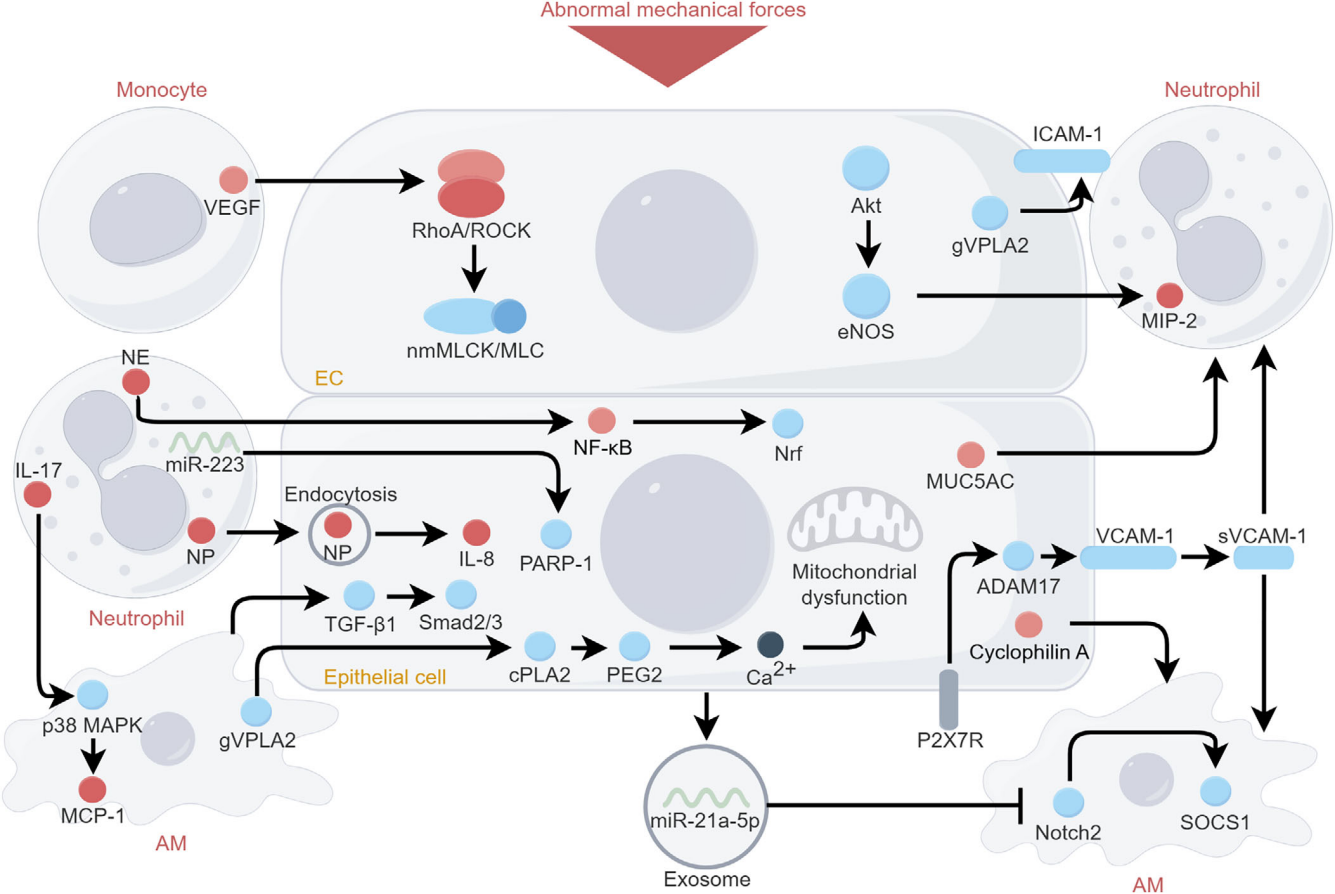
Molecular mechanism of cellular crosstalk in VILI. Cell crosstalk is mediated by direct contact or indirect message delivery. The delivered messages are mainly small molecules or exosome. Immune cells (monocytes, neutrophils, and macrophages) aggravate the dysfunction of stromal cells (ECs and epithelial cells), which in turn activate immune cells and aggravate inflammation. The figure was created by Figdraw (www.figdraw.com).

“Crosstalk” refers to the communication between cells. In the lung, stromal‐immune cell communication maintains homeostasis and responds to injury or infection [183]. The molecular mechanisms of cellular crosstalk are shown in Figure 6.

### 4.1 Endothelial–Epithelial Interactions

Endothelial–epithelial interactions play important role in pulmonary epithelial branching, angiogenesis, and vasculogenesis [184]. VEGF is one of classical molecule for endothelial and epithelial communication [184]. In acute lung injury, epithelial cells regulate endothelial barrier functions through paracrine signaling, while ECs promote alveolar epithelial cell differentiation and injury repair [185]. However, the effects of mechanical stress on endothelial–epithelial interactions, especially in VILI conditions, remain unclear. Future study is supposed to find the mechanism commonality between acute lung injury and VILI, as well as mechano‐dependent special mechanism of interactions.

### 4.2 Immune‐Stromal Cell Communication

ECs are exposed to blood flow, allowing for direct contact with circulating neutrophils. The adhesion between ECs and neutrophils is dependent on the expression of ICAM‐1. HTV MV increases group V phospholipase A2 (gVPLA2) secretion in ECs, which enhances the adhesion between ECs and neutrophils [186]. HTV MV also activated Akt/eNOS pathway in ECs to promote neutrophil sequestration and MIP‐2 release, indicating the effects of ECs on immune cell recruitment and inflammation [144, 187].

Airway epithelial cells subjected to ventilation exhibit an increased expression of mucins, particularly MUC5AC, which exacerbates the accumulation and infiltration of neutrophils [188]. MUC5AC induces inflammation probably because it serves as cytokines “sponge” or promotes the release of ATP [188, 189, 190]. The shedding of soluble VCAM‐1 from ATI serves as a chemoattractant for neutrophils and has the potential to activate AMs [151]. In addition, extracellular cyclophilin A secreted from ATI may be involved in AMs activation and induce VILI [191]. Anti‐inflammatory effects of epithelial cells are also found during ventilation. For example, epithelial‐derived exosomes deliver miR‐21a‐5p to AMs, promoting M2 macrophage polarization through inhibiting Notch2/SOCS1 [192].

Immune cells primarily influence stromal cells through secretory signaling molecules, with direct contact playing a secondary role in their interaction [193]. AMs release gVPLA_2_ activates cytoplasmic phospholipase A_2_/PGE_2_/Ca^2+^ signaling pathway in alveolar epithelial cells to induce mitochondrial dysfunction [194]. The release of NE activates NF‐κB/NRF pathway to promote epithelial cell apoptosis [195], and neutrophil peptides endocytosis by epithelial cells increases IL‐8 production [196]. VEGF released by monocytes increases endothelial leakage [167] likely due to RhoA‐related mechanisms. Moreover, M2 macrophages activate TGF‐β1/Smad2/3 in epithelial cells that contributes to EMT in HTV MV mice [197].

Inflammatory cells also protect stromal cells in VILI, as neutrophils can deliver miR‐223 to epithelial cells, targeting poly (ADP‐ribose) polymerase‐1 to diminish inflammatory response [198]. However, these protective effects are often offset by prolonged and/or harmful ventilation, ultimately failing to prevent exacerbations.

### 4.3 Other Crosstalk

Different types of inflammatory cells activate each other to boost inflammatory process. AMs activation is considered as a quick and early response after ventilation, which further recruit neutrophils [170, 171, 193]. Neutrophil‐derived IL‐17 increases macrophage MCP‐1 expression through p38 MAPK pathway that enhances inflammation in VILI [161]. Other crosstalk in VILI also involves interstitial cell [199] and circulating platelets [200], which modulate EC functions.

### 4.4 Summary and Future Remarks

In VILI, stromal cells play an important role in immune cells trafficking and activation, which relies on the expression of membrane surface receptors and secretion of chemotactic molecules. Immune cells serve as central mediators of inflammation acting both through intrinsic mechanisms and by mobilizing stromal cells. The crosstalk among lung cell types continues to reveal key aspects of VILI pathogenesis and provides new therapeutic perspectives. Furthermore, the interaction between organs reveals mechanisms from a broader macroscopic dimension. Crosstalk between the lungs and other organs, such as the brain [201], heart [202], kidneys [203], and liver [204], attracts increasing attention. This interconnectedness highlights the biological integrity and systemic nature of a whole‐body response to MV and underscores the risks to systemic organs associated with VILI.

## 5 Current and Emerging Therapeutic Strategies

### 5.1 Current MV Strategies

Basic clinical information of VILI is fully reviewed by AK and Anjum in their book [205]. The clinical diagnosis of VILI closely resembles that of ARDS, yet they are not identical conditions. The diagnosis of ARDS involves confirming risk factors, identifying the origin of pulmonary edema, assessing the acute onset of symptoms, conducting chest imaging, and evaluating oxygenation levels [206]. However, the diagnosis of VILI primarily relies on blood oxygenation and chest imaging, while specifically ruling out other potential etiologies that may contribute to these pathological features [205]. The most crucial countermeasure against VILI is the implementation of appropriate ventilatory settings. However, this remains a challenge due to the inherent heterogeneity of ARDS and the individual variability among patients [207]. Current clinical practice guidelines for ARDS from Europe [208], America [209], and Japan [210] recommend lung protective ventilation strategies, and the use of neuromuscular blocking agents presents as an optional consideration. We summarized detailed MV strategies for VILI from these regions in Table 2.

**TABLE 2:** Current clinical practice guidelines for ARDS from Europe, America, and Japan.

	Recommendation	Japanese Society of Intensive Care Medicine/Japanese Respiratory Society/Japanese Society of Respiratory Care Medicine [210]	European Society of Intensive Care Medicine (ESICM) [208]	American Thoracic Society [209]
**Lung protection strategies**	Low tidal volume	Yes, 4–8 mL/kg PBW	Yes, 4–8 mL/kg PBW	Yes, 4–8 mL/kg PBW
	High PEEP	Yes	Unsure	Yes
	Prone positioning	Yes	Yes	Yes
**Drugs**	Neuromuscular blocking agents	Yes	No	Yes

*Note*: “Yes” means the strategy was recommended; “No” means the strategy was opposed; “unsure” means the strategy had no consensus. PBW, predicted body weight.

Recently, mechanical power as a new concept in MV provides a perspective for lung protective ventilation strategies [211]. Mechanical power refers to the amount of energy transferred from ventilator to the respiratory system per unit time, quantifying the complex ventilator parameters into a single value, which optimizes MV strategies in a more intuitive way [211]. Notably, there are no specific pharmacological therapies for VILI in clinical practice, while many classical drugs (such as aspirin, β2 agonists, statins, and keratinocyte growth factor) have been proved ineffective and possibly harmful [212].

### 5.2 Future Therapeutic Prospects

In the future, there are three potential perspectives to improve the clinical conditions of VILI. First, mechanotransduction pathways can be targets to relieve adverse response to abnormal mechanical stress. The therapeutic potential has been proved in animal models, such as targeting to YAP [213] and TRPV4 [35]. Second, biological and regenerative therapies are supposed to be focused, especially stem cell treatment. VILI animals treated with stem cell such as bone marrow‐derived mesenchymal stem cells [214] and endothelial progenitor cells [215] exhibit recovery of barrier permeability and relief of inflammation. Third, biomarkers are used for timely diagnosis and monitoring of VILI conditions, also have potential to become therapeutic targets. In Table 3, we summarized molecules as prospective biomarkers, and evaluated their diagnostic potential based on experiments. Then we searched drugs targeted to these biomarkers in Drugbank (go.drugbank.com), to provide alternative options for future pharmacological treatments.

**TABLE 3:** The molecules as prospective biomarkers and/or therapeutic targets of VILI.

Molecules	Detectable source	Variation	Species	Diagnostic potential	Targeted drugs	Drug development stage
CD14 [216]	Lung tissue	Increased	Rabbit	Yes	Atibuclimab	Investigational
Cellular communication network factor 1 (CCN1) [217]	BALF, lung tissue	Increased	Human, mouse	Yes	No	—
Platelet‐endothelial cell adhesion molecule‐1 (PECAM1) [218]	Plasma, lung tissue	Increased in plasma, decreased in lung tissue	Rat	Yes	No	—
Neutrophil gelatinase‐associated lipocalin (NGAL) [219]	Plasma, BALF, lung tissue	Increased	Mouse	Yes	Methyl nonanoate, 2,3,‐Dihydroxybenzoylserine, Trencam‐3,2‐Hopo, 2,3‐Dihydroxy‐Benzoic Acid, Carboxymycobactin S, Carboxymycobactin T	Experimental
Exhaled pentanal [220]	Breath	Increased	Rat	Yes	No	—
CXCL10/CXCR3 [221]	Plasma	Increased	Human, rat	Yes	Clove oil	Approved/nutraceutical
Clara cell protein (CC16) [222]	Plasma, pulmonary edema fluid	Decreased	Human	Yes	No	—
IL‐10 [223]	—	—	Rat	No	CRx‐139 (TNF, IL‐6, C‐reactive protein), LLL‐3348	Experimental
Resolvin D1 [224]	—	—	Mouse	No	No	—
TRIM72 [225]	—	—	Mouse	No	No	—
Cyclooxygenase (COX)‐2 [226]	Prostaglandin (PG)E2 and 6‐keto PGF1a in BALF, lung tissue	Increased	Mouse	Yes	Robenacoxib, SC‐236 (NF‐κB), NS‐398	Vet approved/ experimental
HMGB1 [70, 157, 227]	Plasma, BALF, lung tissue	Increased	Rabbit, mouse, rat	Yes	Ethyl pyruvate (TNF), Chloroquine (TNF, TLR9)	Approved/investigational
Hyaluronan Synthase 3 [228]	Lung tissue	Increased	Mouse	Yes	No	—
IL‐6 [229]	BALF, lung tissue	Increased	Mouse	Yes	Ginseng, Atiprimod (TNF, STAT3), Siltuximab, Olokizumab, YSIL6 (TNF), VX‐702 (IL‐1β, TNF, MAPK14), Foreskin keratinocyte (neonatal) (other 11 targets), CRx‐139 (IL‐6, TNF, C‐reactive protein), Andrographolide (TNF, IL‐1β, NF‐κB), Polaprezinc, Foreskin fibroblast (neonatal) (other 10 targets), Dilmapimod (TNF, IL‐1β, MAPK14)	Approved/investigational/experimental
Inos [230, 231]	Lung tissue	Increased	Mouse, rat	Yes	46 kinds of drugs	—
Inositol 1,4,5‐trisphosphate receptor (IP3R) [232]	Lung tissue	Increased	Mouse	Yes	Caffeine (other 14 targets)	Approved/investigational
Plasminogen activator inhibitor (PAI)‐1 [233]	Plasma, lung tissue	Increased	Rat	Yes	10 kinds of drugs	—
miR‐214 [234]	Lung tissue	Increased	Mouse	Yes	No	—
TNF [235]	BALF	Increased	Mouse	Yes	36 kinds of drugs	—
WNT1‐inducible signaling pathway protein 1 (WISP1) [236]	BALF, lung tissue	Increased	Mouse	Yes	No	—

*Note*: “Yes” means the molecule has diagnostic potential (based on experiments) for VILI; “No” means the molecule lacks evidence for its diagnostic potential for VILI, or has no targeted drugs. The brackets following the drug names indicate their other binding targets (data from Drugbank, go.drugbank.com).

In terms of clinical trials, few researchers focus on the pharmacological treatment of VILI, perhaps due to varied pathological conditions and unclear targets. In Table 4, we summarized several clinical trials of pharmacological treatment to VILI in ClinicalTrials (clinicaltrials.gov).

**TABLE 4:** Clinical trials of pharmacological treatment to VILI.

Target	Intervention/treatment	Related mechanisms	Phase	Current status	ClinicalTrials.gov identifier
eNAMPT	ALT‐100 mAb	Inflammation relief	2	Active, not recruiting	NCT05938036
sα2B adrenergic receptors	Centhaquine	Hemodynamic recovery	2	Not yet recruiting	NCT05241067
Nerves and muscles	Sevoflurane	Sedation	3	Completed	NCT04235608

## 6 Knowledge Gaps and Future Directions

### 6.1 Need for Better Mechanobiology Models

In vitro, the two‐dimensional growth environment and oversimplified culture conditions result in inherent limitations compared to in vivo situations, such as the absence of ECM interactions and communications with other cell types during stretch application. Perhaps more importantly, the usual biomechanical environments of cell culture are unphysiologically stiff which we consider limits the predictive pharmacology of findings made in these settings [237]. The FDA modernization Acts 2.0 and 3.0 are encouraging the adoption non‐animal methods in the drug regulatory path [238]. The microphysiological systems (MPS) approach to investigation of VILI is in its infancy but holds promise through its focus on the use of human cells, avoiding species restricted findings in rodent models that limit clinical translational impact. Devices such as Emulate's flexible epithelial endothelial stretching lung‐on‐a‐chip environment or more bespoke designs such as the micro‐scale humanized ventilator on a chip [239] are offering better physiological emulation. In vitro systems typically employ atmospheric oxygen levels whereas ventilation is often accompanied by oxygen supplementation. Moreover, in vitro studies typically use a cell culture media that has amino acid and glucose concentrations may fold higher than found in plasma or interstitial fluids. These additional considerations in efforts to emulate VILI will likely yield different outcomes, as they have in studies on anti‐cancer drugs [240].

In vivo, VILI models using healthy individuals ignore clinical settings, as patients requiring MV usually present with underlying medical conditions [241, 242]. Furthermore, animal VILI models often employ highly pathogenic parameters such as high total volumes and short ventilation durations [95, 243], and many relevant signaling pathways are predominantly activated under such pathological (non‐physiological) conditions [112, 244].

### 6.2 Multi‐Dimensional and Multi‐Omics Analysis

Species differences, variability in experimental parameters, and divergent research environments sometimes yield opposite results, indicating the need for clinical translation to focus on spatio‐temporal dynamics and individual variability. More advanced preclinical models, such as multiple‐hit models, organoids, and organ‐on‐a‐chip systems, require further development. As a relatively upstream process, mechanotransduction is difficult to correlate directly with specific ventilator settings. Instead, easily detectable biomarkers are needed to enable early identification, timely intervention, and dynamic adjustment of ventilation strategies in VILI. Single‐cell sequencing and multi‐omics approaches enable in‐depth, multi‐dimensional analysis of molecular mechanisms, often revealing new perspectives and potential therapeutic targets [245, 246]. In clinical practice, artificial intelligence is increasingly employed to integrate complex information, including MV parameters, laboratory results, demographic data, and admission reasons, enabling the development of mixed neural network models for real‐time predictive adjustment of ventilation parameters [247].

### 6.3 Personalized MV

It is essential to emphasize inter‐patient variability and individualized parameter thresholds. For obese patients, ventilation presents greater challenges and risks, thus pre‐oxygenated in a head‐up position (not less than 30°) with a high inspiratory fraction of oxygen is recommended along with preparedness for rescue strategies in case of difficulty [241]. Patients with interstitial lung disease should be applied to strict low tidal volume (4–6 mL/kg) and minimal PEEP to avoid overdistension [242]. For pediatric ARDS patients, a more conservative strategy is recommended, emphasizing personalized PEEP titration and prioritizing non‐invasive ventilation [248]. Furthermore, greater consensus on ARDS subtypes, such as pneumonic versus non‐pneumonic, is needed in the future, along with further exploration of the potential of rheological model to define individualized thresholds [249].

## 7 Conclusion

VILI is an iatrogenic consequence of MV. The original life‐support strategies for respiratory failure often come with accompanying risks that can exacerbate the patient's condition. Improvements in clinical practice regarding MV, including careful monitoring of airway pressure and adjustment of tidal volume and PEEP, have significantly ameliorated VILI compared to previous decades. However, the risk of injury associated with ventilation remains a pressing clinical challenge, as the diversity of patients and the variability in ARDS complicate the establishment of uniform standards for care.

**FIGURE 7:**
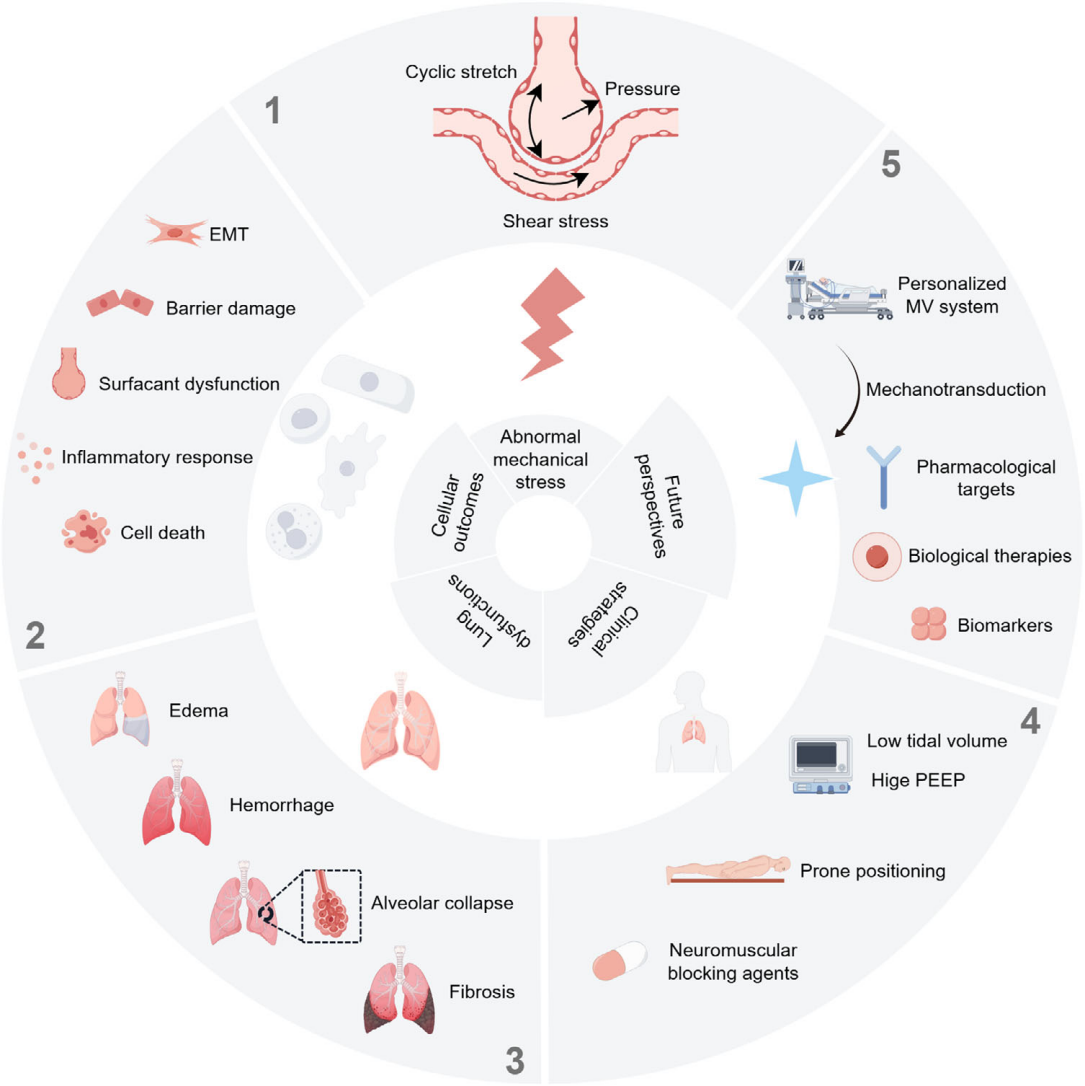
Envisioning mechanobiology‐based therapies for VILI. A schematic model to illustrate the hierarchical progression of mechanotransduction—from abnormal mechanical stimuli, cellular outcomes, lung dysfunctions, clinical strategies, and future perspectives. The figure was created by Figdraw (www.figdraw.com).

MV is expected to undergo significant transformative advancements by 2050, with the emergence of personalized ventilation systems that promise to revolutionize respiratory care. The future MV system will enable precise optimization of ventilation parameters tailored to each patient's unique physiological profile and clinical status [250]. However, the risk of VILI remains a significant concern at this stage. Our unpublished single‐cell sequencing results show that even with the optimized ventilator parameters, it will still cause a certain degree of pulmonary damage. Therefore, in addition to continuously improving MV strategies, it is crucial to explore the molecular mechanisms underlying VILI. Notably, investigating the mechanobiological mechanism of VILI will also help improve the ventilation systems. Figure 7 summarizes the hierarchical progression of VILI, including pathogenic mechanism (mechanical stimuli, mechanotransduction, and cellular outcomes), current clinical strategies and future treatment directions.

## Author Contributions

H.R. did the literature search, visualization, writing, and prepared the original draft, reviewing, and editing. Z.S. performed visualization, writing, prepared the original draft. A.S. did the writing, reviewing, editing, supervision, and funding acquisition. Y.Q. did writing, review, editing, supervision, project administration, and funding acquisition. K.H. did the conceptualization, prepared the original draft, writing, reviewing, editing, project administration, and funding acquisition. All authors and their contributions have been mentioned and all authors have read and approved the final manuscript.

## Funding

This research was supported by grants from the National Natural Science Foundation of China (Grant Numbers: 12032003 and 12302409), and Australian Research Council (Grant Number: IC25100027).

## Ethics Statement

The authors have nothing to report.

## Conflicts of Interest

The authors declare no conflicts of interest.

## Data Availability

The authors have nothing to report.
